# Deleterious consequences of antioxidant supplementation on lifespan in a wild-derived mammal

**DOI:** 10.1098/rsbl.2013.0432

**Published:** 2013-08-23

**Authors:** Colin Selman, Jane S. McLaren, Andrew R. Collins, Garry G. Duthie, John R. Speakman

**Affiliations:** 1 Institute of Biological and Environmental Sciences, University of Aberdeen, Aberdeen AB24 2TZ, UK; 2 Rowett Institute of Nutrition and Health, University of Aberdeen, Aberdeen AB21 9SB, UK; 3 State Key Laboratory of Molecular Developmental Biology, Institute of Genetics and Developmental Biology, Chinese Academy of Sciences, Chaoyang 100100, Beijing, People's Republic of China

**Keywords:** oxidative damage, ageing, longevity, vitamin E, vitamin C, vole

## Abstract

While oxidative damage owing to reactive oxygen species (ROS) often increases with advancing age and is associated with many age-related diseases, its causative role in ageing is controversial. In particular, studies that have attempted to modulate ROS-induced damage, either upwards or downwards, using antioxidant or genetic approaches, generally do not show a predictable effect on lifespan. Here, we investigated whether dietary supplementation with either vitamin E (α-tocopherol) or vitamin C (ascorbic acid) affected oxidative damage and lifespan in short-tailed field voles, *Microtus agrestis*. We predicted that antioxidant supplementation would reduce ROS-induced oxidative damage and increase lifespan relative to unsupplemented controls. Antioxidant supplementation for nine months reduced hepatic lipid peroxidation, but DNA oxidative damage to hepatocytes and lymphocytes was unaffected. Surprisingly, antioxidant supplementation significantly shortened lifespan in voles maintained under both cold (7 ± 2°C) and warm (22 ± 2°C) conditions. These data further question the predictions of free-radical theory of ageing and critically, given our previous research in mice, indicate that similar levels of antioxidants can induce widely different interspecific effects on lifespan.

## 1. Introduction

Until recently, the most dominant mechanistic theory of ageing was the free-radical theory of ageing (FRTA), now more commonly termed the oxidative damage theory of ageing [1,2]. Essentially, it suggests that damage to proteins, lipids and DNA is caused by free radicals, more specifically reactive oxygen species (ROS), primarily generated as a by-product of mitochondrial oxidative phosphorylation. Despite the existence of an extensive array of defence and repair systems to prevent and mitigate damage [1,3–5], the FRTA predicts that a proportion of ROS evade these systems, leading to oxidative damage. This damage consequently accumulates with age, initiating a series of events that over the time compromise function, leading to pathology and ultimately death [1,6]. However, despite extensive correlative evidence supporting the FRTA, several recent studies have raised reservations over the role of ROS in causing ageing [6–8]. The idea that supplementation with antioxidants such as vitamins E and C can decrease ROS and oxidative damage, and hence increase lifespan is pervasive, despite a lack of convincing supportive data [9]. We previously reported that lifelong dietary vitamin E supplementation in laboratory mice (C57BL/6) extended lifespan relative to controls, although oxidative damage to lipids and DNA was unaffected [10]. Vitamin C supplementation in the same mouse strain had no effect on lifespan or oxidative damage, but did reduce expression of several genes linked to antioxidant protection [11]. The effects of antioxidant supplementation on disease and mortality in humans are equally ambiguous [12], with some meta-analysis approaches even suggesting that mortality is increased following supplementation of certain antioxidants [13,14].

Given this confusion surrounding the FRTA, there has been a recent drive to understand the role of ROS-induced oxidative damage in shaping life histories and to examine the relationship between oxidative damage and ageing in non-model organisms [3,4,15]. To this end, we examined lifespan and oxidative damage in short-tailed field voles (*Microtus agrestis*) supplemented from two months of age with either dietary vitamin C (ascorbyl-2-polyphosphate) or dietary vitamin E (α-tocopherol), relative to unsupplemented controls. Voles were supplemented at identical concentrations to our previous mouse studies [10,11]. We predicted that metabolic rate, ROS production and oxidative damage would be elevated under cold conditions in rodents, thus resulting in a greater likelihood of detecting the effect of antioxidant supplementation on oxidative damage and lifespan [10,11]. Consequently, we maintained both control- and antioxidant-supplemented voles at housing temperatures of either 7 ± 2°C (cold) or 22 ± 2°C (warm).

## 2. Material and methods

Short-tailed field voles were collected from a wild population and maintained under experimental conditions as previously described [16]. At approximately one month of age, an individual from the same sex sibling pair was transferred to the cold (7 ± 2°C), while the other one remained in the warm (22 ± 2°C). At two months of age, animals at each temperature were randomly assigned to either a control diet (RM1 diet containing 10 mg kg^−1^ ascorbyl-2-polyphosphate and 22 mg kg^−1^ α-tocopherol), a vitamin C-supplemented diet (RM1 + 180 mg kg^−1^ of ascorbyl-2-polyphosphate) or a vitamin E-supplemented diet group (RM1 + 550 mg kg^−1^ α-tocopherol). The comparison of the warm and cold unsupplemented animals used as controls here was reported previously elsewhere [16]. All lifespan experiments were run simultaneously, with Kaplan–Meier survival curves constructed using known birth and death dates of each individual, with *p-*values calculated using the log-rank test [16]. We compared the effects of the two supplemented diets using a mixed-effects model with diet and temperature as fixed effects and sibling pair as a random effect nested within diet. An additional cohort was used to examine the effects of antioxidant supplementation on food intake, body mass and oxidative damage at 11 months of age (nine months supplementation). Hepatic lipid peroxidation was estimated by measuring thiobarbituric acid reactive substances using high-performance liquid chromatography [10,11,16]. Hepatic and lymphocyte DNA oxidative damage was determined using the modified Comet assay [10,11,16], which uses the lesion-specific bacterial repair enzymes endonuclease III (ENDO III) and formamidopyrimidine-DNA glycosylase (FPG) to increase sensitivity and specificity. ENDO III induces breaks at oxidized pyrimidine sites and FPG induces breaks at purines, including 8-oxo-guanine [17]. Sample sizes are reported in the electronic supplementary material, table S1. Data were deposited in the Dryad repository: http://dx.doi.org/10.5061/dryad.31cc4 [18].

## 3. Results

**Figure 1.**
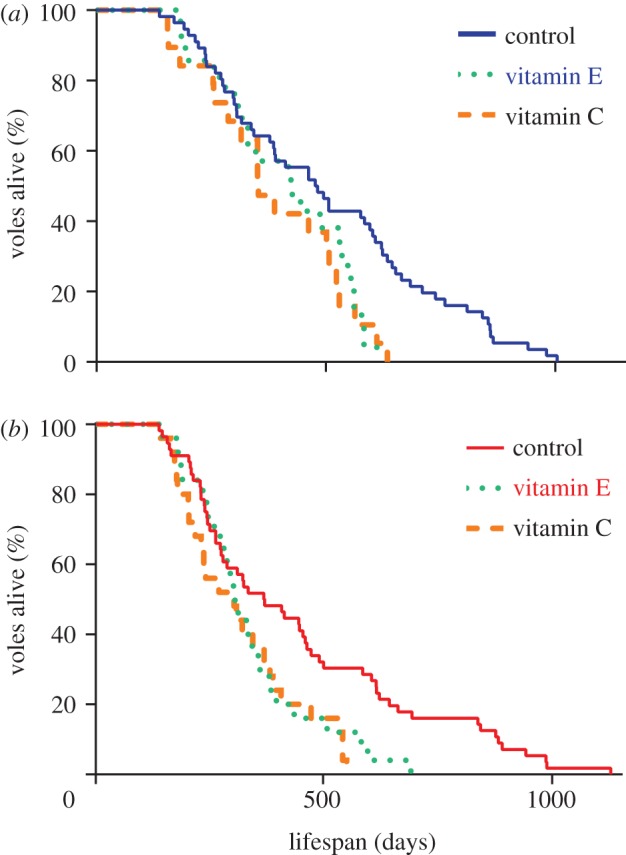
Kaplan–Meier survival curves of voles maintained in the cold (*a*) 7 ± 2°C or warm (*b*) 22 ± 2°C and given access to either a control diet, a vitamin E-supplemented diet or a vitamin C-supplemented diet from two months of age. (*a*) Solid blue line denotes control, stippled green line denotes vitamin E and broken orange line denotes vitamin C groups. (*b*) Solid red line denotes control, stippled green line denotes vitamin E and broken orange line denotes vitamin C groups.

**Figure 2.**
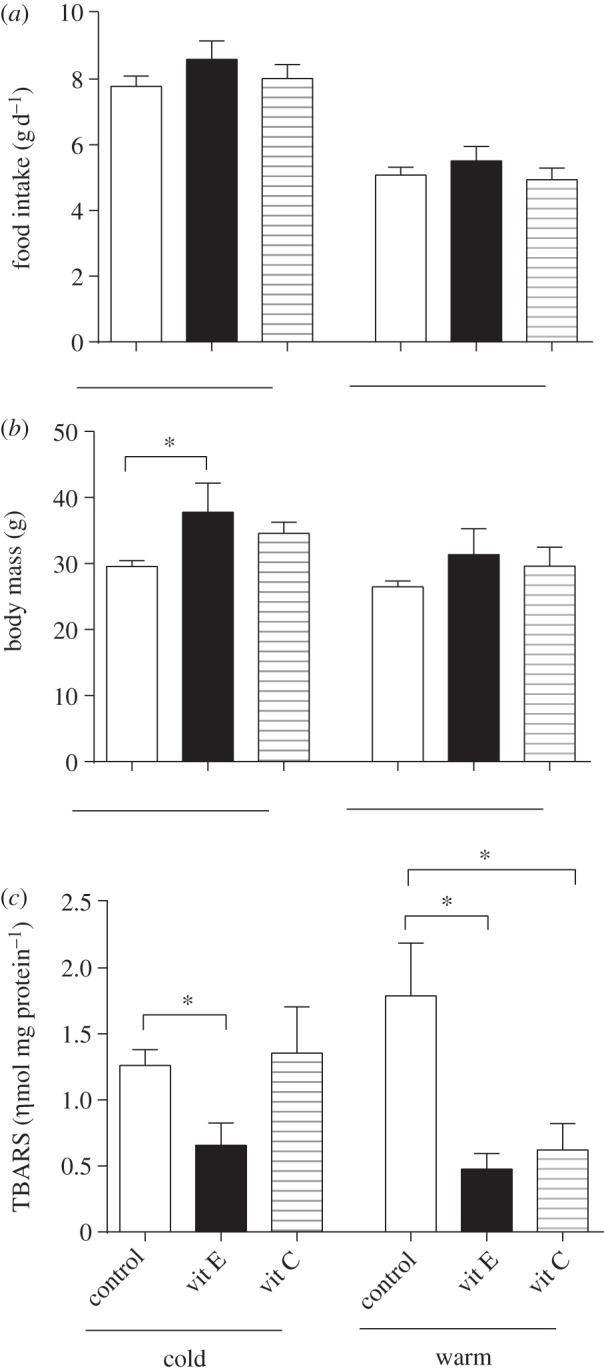
Mean (±s.e.m.) daily food intake ((*a*) g d^−1^), body mass ((*b*) g) and hepatic lipid peroxidation ((*c*) ηmol mg protein^−1^) levels at 11 months of age in vitamin E- and vitamin C-supplemented voles maintained in the cold and warm relative to their respective unsupplemented controls. Asterisk (*) denotes significant difference to appropriate control where *p* < 0.05.

Voles maintained on the control diet in the cold (figure 1*a*) lived significantly longer than those fed either the vitamin E (log-rank test, *X*_2_ = 5.804, *p* = 0.016) or vitamin C diets (*X*_2_ = 6.052, *p* = 0.014), with median lifespan being 477, 424 and 353 days, respectively. Similarly, control voles in the warm (figure 1*b*) lived significantly longer than the vitamin E (*X*_2_ = 5.008, *p* = 0.025) or C-supplemented animals (*X*_2_ = 7.588, *p* = 0.006), with median lifespan being 368, 305 and 303 days, respectively. When comparing the effects of the different supplemented diets, we found no effect of diet (*F*_1,59_ = 0.04, *p* = 0.834), no effect of individual sibling pair (*F*_28,59_ = 0.97, *p* = 0.535) and no significant interaction of diet and temperature (*F*_1,29_ = 0.16, *p* = 0.689). However, there was a significant effect of ambient temperature (*F*_1,59_ = 5.32, *p* = 0.029) on lifespan, with supplemented individuals in the cold living longer on average (391 days) compared with individuals in the warm (307 days). Daily food intake (figure 2*a*) at 11 months of age did not differ between control- and antioxidant-supplemented voles either in the cold (*F* = 0.949, *p* = 0.390) or in the warm (*F* = 2.382, *p* = 0.101). However, a significant difference in body mass (figure 2*b*) was observed between groups in the cold (*F* = 5.383, *p* = 0.007) at this time, with vitamin E-supplemented voles being significantly heavier than the control voles (post hoc Tukey test, *p* = 0.017). No difference in body mass was detected between animals maintained in the warm (figure 2*b*; *F* = 1.755, *p* = 0.182). Hepatic lipid peroxidation (figure 2*c*) was significantly lower in vitamin E-supplemented mice relative to controls in the cold (*t* = 2.986, *p* = 0.011) and warm (*t* = 2.513, *p* = 0.029). Vitamin C supplementation also reduced lipid peroxidation relative to controls in the warm (figure 2; *t* = 2.348, *p* = 0.037), but not in the cold (*t* = 0.272, *p* = 0.790). Lymphocyte (see the electronic supplementary material, figure S1*a* and S1*b*) and hepatocyte (see the electronic supplementary material, figure S2*a* and S2*b*) DNA oxidative damage was generally elevated in the supplemented groups at both housing temperatures, although this did not reach any statistical significance for comparison (*p* > 0.05; exact *p-*values reported in the electronic supplementary material).

## 4. Discussion

In contrast with our previous findings on laboratory mice [10,11], our data clearly demonstrate that dietary supplementation with either vitamin E or vitamin C dramatically shortened lifespan in voles. This occurred despite the fact that hepatic lipid peroxidation was significantly reduced in all but one (cold, vitamin C-supplemented) treatment group, although lymphocyte and hepatocyte DNA oxidative damage was unaffected by antioxidant supplementation. The reasons for this lifespan effect are currently unclear. Dietary restriction extends lifespan in many animals [19], and the antioxidant diets may have been more palatable, driving hyperphagia that potentially affect health and survival. While absolute daily food intake at 11 months of age was unaffected by diet, supplemented voles at both temperatures tended to be heavier than the control animals, although this reached significance, relative to controls, only in the cold exposed vitamin E group. Increased body mass is a major risk factor for many pathologies [20], hence it would be interesting to know how our antioxidant diets impacted on metabolism and overall health. In addition to a negative impact of antioxidants on lifespan, voles fed the antioxidant-supplemented diets in the cold (where metabolic rate is increased [16]) lived approximately 27 per cent longer than those in the warm. This effect could be mediated via elevated uncoupling of metabolic rate in the cold conditions which has been previously implicated as a factor influencing lifespan [21–24]. Both the negative impact of antioxidant supplementation and positive effect of cold exposure on lifespan cast further doubt on simplistic models relating metabolism, ROS production and ageing [4,6–8]. Antioxidants elicit many cellular effects that are unrelated to ROS-quenching *per se* [25,26], and it is undoubtedly naive to think that other pathways and processes are not affected following supplementation. For example, the positive effects of exercise on insulin sensitivity in humans are lost when antioxidants are given [27], vitamin C can act as a pro-oxidant under certain conditions [28], and in yeast, at least, vitamin E supplementation (*α*-tocopherol) has been shown to increase ROS production, increase oxidative damage and decrease lifespan [29]. In addition, antioxidant efficacy in quenching ROS *in vitro* appears not to be predictive of lifespan in *Caenorhabditis elegans* [30]. We did not measure ROS directly or determine endogenous antioxidant levels, hence cannot be certain that antioxidant supplementation did not affect other aspects of antioxidant protection, as seen in mice where lifelong vitamin C supplementation reduced various parameters associated with antioxidant protection [11]. However, it should be noted that this dampening of endogenous antioxidant protection in mice did not have any impact on lifespan [11]. What our findings do indicate is that significant variation exists in the effects of antioxidants on oxidative damage and lifespan across species. We suggest that there is a need for further comparative studies in this area, and that caution should be used when advocating that antioxidants might protect against oxidative damage and ageing in humans, based on studies of limited numbers of species.
